# Investigating the draw ratio and velocity of an electrically charged liquid jet during electrospinning

**DOI:** 10.1039/c9ra02024a

**Published:** 2019-05-02

**Authors:** Chenhui Ding, Hong Fang, Gaigai Duan, Yan Zou, Shuiliang Chen, Haoqing Hou

**Affiliations:** College of Chemistry and Chemical Engineering, Jiangxi Normal University Nanchang 330027 China haoqing@jxnu.edu.cn +86 791 8812 0389; College of Materials Science and Engineering, Nanjing Forestry University Nanjing 210037 China duangaigai@njfu.edu.cn +86 25 85428090; Department of Mechanics, Huazhong University of Science and Technology Wuhan 430074 China

## Abstract

The investigation of the draw ratio and velocity of an electrospinning polymer solution jet is of great interest for understanding the formation of nanofibers. During the electrospinning process, the charged polymer solution jets were stretched by electric force, resulting in the formation of ultrathin fibers. In this study, theoretical deduction and experimental calculation were applied to evaluate the velocities and draw ratios of the charged jets at different electrospinning stages. Depending on the diameter of the charged jets at different electrospinning stages, the velocities and draw ratios of the charged jets were calculated with values far lower than the data in a previous report. The theoretical calculation was compared with experimental data using polyamic acid as a model polymer for electrospinning. The results indicated that during electrospinning, as the collecting distance was increased from 0 to 30 cm, the diameter of the electrospinning jet decreased from 18 800 nm to a constant value of around 245 nm, the solvent in the jet decreased from 96.50 wt% to 25.45 wt%, and the density of the jet increased from 0.9504 to 1.0995 g cm^−3^. These parameters led to the draw ratio and velocity of the jet experiencing first an increase and then a decrease in the value, and the highest draw ratio and velocity were 869 and 867 m s^−1^, respectively, which are quite different from the data in previous reports.

## Abstract 2

Theoretical calculations and experiments were performed to determine the draw ratio and velocity of an electrospinning jet.

## Graphical abstract

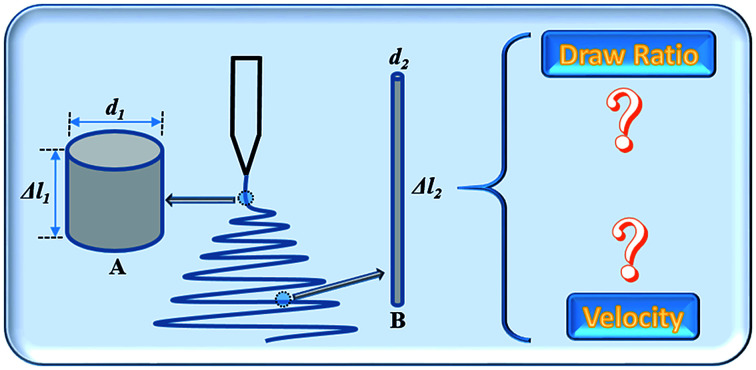

## 1. Introduction

As one of the effective technologies for producing fibers with an ultrathin fiber diameter, electrospinning has attracted much attention recently^1,2^ due to new perspectives for numerous applications in a broad range of areas, such as tissue engineering,^3,4^ heavy metal detection,^5^ filtration,^6–10^ oil/water separation,^11–14^ catalysts,^15–19^ sensitive and responsive materials,^20–24^ nanofiber reinforced composites,^25,26^ high efficiency electrodes in electrochemical cells,^27–30^ heat resistant materials,^31^ white light-emitting materials,^32,33^ and sponge materials.^34–36^ During the electrospinning process, an electrically charged jet is created from a pendant droplet of polymer solution. After forming a nearly straight line, it is bent into a complex path and other changes in shape occur under the stretch of electric forces. Surprisingly, the liquid jet can be drawn in a strong electric field during the electrospinning process.^37–40^ Polymer chains in electrospun polymer fibers are oriented under the induction of an electric field.^41^ The orientation of the polymer chains depends on the draw ratio.^42^ It is very important to investigate the draw ratio of the electrospinning charged jet because the draw ratio will significantly affect the molecular orientation, and therefore influence the physical properties, especially the mechanical properties.^43–46^ There are some studies aimed at deducing the velocity of jet and the draw ratio.^38,47–53^ However, because of the complexity of the electrospinning process, too many electrospinning parameters, such as polymers, concentration, evaporation of solvent, flow rate, should be considered, which leads to the difficulties in proposing a universal model to deduce the draw ratio and velocity of the electrospinning jet. For example, in a very early study, the draw ratio of electrospun fibers was evaluated using an aqueous solution of polyethylene oxide (PEO).^38^ In that study, the starting diameter of a PEO solution jet with a concentration of 6% was assumed to be 100 μm and the final diameter of the fibers was 100 nm. Then, the draw ratio would be 10^6^ without accounting for the evaporation of solvent. If considering the evaporation of the solvent, the draw ratio would be 0.06 × 10^6^ = 60 000. Furthermore, the velocity at the nanofiber end of the jet was deduced to be about 60 000 m s^−1^, which was 176 times faster than the speed of sound in air, which is more than five times faster than the speed that objects need to get out of the Earth's atmosphere (second cosmic velocity = 11 200 m s^−1^).^38^ However, it is well-known that the draw ratio of conventional fibers is in the range of 5 to 40.^54^ Even for the ultra-high molecular weight polyethylene fibers, the draw ratio is merely 35 0,^55^ which is much lower than the draw ratio in a previous report.^38^ In another recent research, Zheng *et al.* developed a model to simulate the dynamic processes that occurs during electrospinning.^47^ Their simulation demonstrated that when the simulation voltage was 5 kV, the maximum velocity was about 2 m s^−1^; which was quite inconsistent with 60 000 m s^−1^.

Therefore, it is still necessary to develop a new way to evaluate the draw ratios and velocities of charged jets during electrospinning. In the present study, the draw ratio and velocity of jet were deduced based on the mass conservation of the polymer in the initial selected jet with a certain length. The mass of the polymer in the initial selected length remains constant even when the selected jet is elongated at different electrospinning times. Therefore, the model does not need to consider all of the complex electrospinning parameters but to consider the concentration and the diameter of the jet, which can be easily obtained as shown in the following sections. To show the application of the model in this study, a typical example is applied, in which the polyamic acid (PAA) is electrospun into nanofibers. The corresponding draw ratios and velocities of the charged jets during electrospinning are also calculated.

## 2. Materials and methods

### 2.1. Materials

3,3′,4,4′-Biphenyltetracarboxylic dianhydride (BPDA) was supplied by Hebei Jida Plastic Products Co., China and 4,4′-diaminodiphenyl ether (ODA) was bought from Quzhou Kaiyuan Fine Chem. Co., China. These two monomers were purified by sublimation before use. *N*,*N*′-Dimethylacetamide (DMAc, 99%) was supplied by Tianjin Fu Chen Chemical Reagent Factory, China. It was used as received.

### 2.2. Preparation of polyamic acid (PAA) solution

The precursor of polyimide, polyamic acid (PAA) of BPDA/ODA was synthesized from an equal molar of dianhydride BPDA with diamine ODA. The polycondensation was performed in DMAc at −4–0 °C for 24 h and the solid contents of PAA solution was maintained to 10 wt%. The intrinsic viscosity of PAA was 4.5 dL g^−1^.

### 2.3. Electrospinning

Before electrospinning, the 10 wt% PAA solution was diluted to 3.5 wt% by DMAc. During electrospinning, the voltage applied to the pendant drop and grounded collector was set to +20 kV and −5 kV. The flow rate was set to 1 mL h^−1^. The temperature and humidity for electrospinning were 20 °C and 30%, respectively. PAA nanofibers were collected by a water bath with different collecting distances. The obtained PAA fibrous mats were dried in a vacuum oven at 70 °C for 24 h to completely remove the solvent inside. The weights of the PAA fibrous mats at different collecting distances were measured before and after drying to give the concentration of polymers in the fibers.

### 2.4. Characterization

A scanning electron microscope (SEM, FEI Quanta 200 FEG) was used to observe the morphology of the electrospun fibers. The fiber diameter was measured using the Image J software. The intrinsic viscosity of PAA was measured by an Ubbelohde viscosity meter at 25 °C using DMAc as solvent.

## 3. Results and discussion

### 3.1. Symbols used in this work

In this study, many symbols are used. Their definitions and corresponding units are listed in Table 1.

**Table 1:** Symbols employed and their definitions

Symbol	Definition	Unit
*S*_1_	Cross-sectional area of straight segment	μm^2^
*S*_2_	Cross-sectional area of final nanofiber jets	μm^2^
Δ*l*_1_	Differential length of straight segment jet	nm
Δ*l*_2_	Differential length of charged bending fiber jet	nm
*υ*_1_	Velocity of straight segment of jet	m s^−1^
*υ*_2_	Velocity of charged bending fiber jet	m s^−1^
*Q*_s_	Consumption of spinning solution	mL
*Q*_p_	Consumption of polymer	g
*V*_s_	Flow rate of spinning solution	mL h^−1^
*t*	Electrospinning time	h
*d*_1_	Diameter of the straight segment of jet	μm
*d*_2_	Diameter of charged bending fiber jet	nm
*C*_1_	Concentration of polymer solution	
*C*_2_	Percentage of polymer in bending fiber jet	
*ρ*_1_	Density of straight segment of jet	g cm^−3^
*ρ*_2_	Density of charged bending fiber jet	g cm^−3^
*@*	Draw ratio	

### 3.2. Diameter in the straight segment of jets

**Fig. 1:**
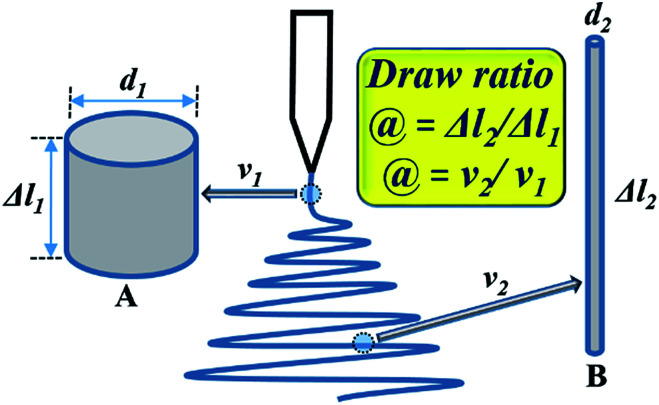
Schematic of an electrospinning jet model. (A) Straight segment and (B) charged bending fiber jet.

In an electrospinning process, the pendant drop of polymer solution is first stretched into a straight linear part with a length of several millimeters to a few centimeters due to the electrical force, then it is bent and forms a loop-shaped fiber jet, as shown in Fig. 1. If the jet including the linear part and the loop-shaped part is an uninterrupted single jet and the electrospinning solution is only from the single jet, then the consumption of spinning solution is equal to the volume of spinning solution. Denoting the cross-sectional area of the straight segment of charged jet by *S*_1_, velocity by *υ*_1_, spinning time by *t*, the consumption of spinning solution *Q*_s_ is given by:

1 *Q*_s_ = *υ*_1_*tS*_1_

The flow rate of the solution, *V*_s_, is therefore:2
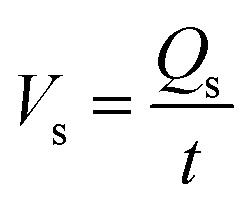


Combining eqn (1) and (2), and accounting for the fact that *S*_1_ = π(*d*_1_/2)^2^, we obtained eqn (3) for the initial diameter of the jet,3
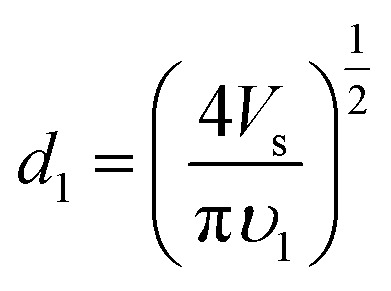


If the velocity along the straight segment *υ*_1_ is known, then it is possible to calculate the diameter *d*_1_. Warner *et al.*^56^ measured the velocities along the straight segment *υ* using laser Doppler velocimetry and reported that the velocity ranged from 1 to 15 m s^−1^. Therefore, according to eqn (3), the diameter of the straight segment decreases from 18.8 *V*_s_^1/2^ to 4.9 *V*_s_^1/2^ μm. The diameter of the straight jet is large at the beginning and small at the end.

### 3.3. Draw ratio of jet in electrospinning

In the previous section, the diameter of the straight segment jet can be easily calculated, varying from 4.9 *V*_s_^1/2^ to 18.8 *V*_s_^1/2^ μm. Here, we assume that the flow rate of the spinning solution *V*_s_ is 1 mL h^−1^, so the biggest diameter is 18.8 μm. When the flow rate *V*_s_ reaches up to 2 mL h^−1^, the diameter is no more than 26.6 μm, which is still much less than that of 100 μm estimated in a previous report.^38^

Electrospun fibers experience quite a different fiber drawing process from the conventional dry-spinning fibers. The dry-spinning process begins with one solid state and ends with the other solid state. The densities of the two solid states are different, but the quantity of the solid remains the same. However, due to the very fast evaporation and removal of solvent in electrospinning, there is a change in the quantity from a liquid jet to solidified fibers. So, the mass of polymer in the two parts is equal,4
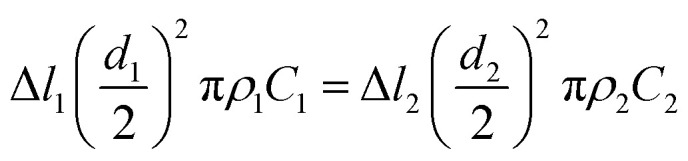
where Δ*l*_1_ and Δ*l*_2_ represent the differential lengths of the initial jet and solidified nanofibers, respectively, *d*_2_ is the diameter of the nanofibers, *ρ* is the density of the charged jet, and *C* is the concentration of jets. Then, the draw ratio *@* can be expressed as follows:5
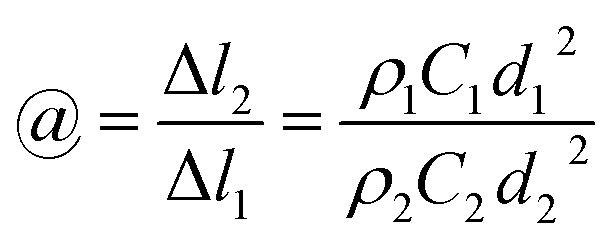


Combining this expression with eqn (3) gives6
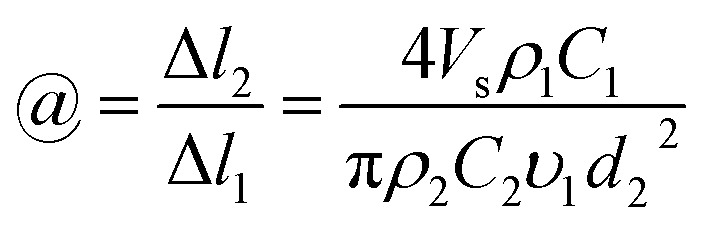


In a previous study,^38^ the mass concentration of 6% PEO was treated as a volume mass concentration, which is defined as the weight of the solute in a 100 mL solution. The flow rate of the spinning solution is 1 mL h^−1^, the velocity of jet in a straight segment is 1 m s^−1^, assuming that the density of the PEO nanofibers was 1.3 g cm^−3^. According to eqn (6), the draw ratio is 1632 and the final velocity of the PEO nanofiber jet achieved is only 1632 m s^−1^, which is far below the draw ratio (60 000) and the velocity (60 000 m s^−1^) in the previous report.^38^

### 3.4. Estimation of jet velocity

The investigation of the velocity of jet is to estimate the draw ratio during the electrospinning process and obtain information about the macromolecular chain orientation. The draw ratio in eqn (6) is deduced from one point of the initial jet to the other point of solidified jet. The velocity of the nanofiber jet *υ*_2_ (Δ*l*_2_ = *υ*_2_*t*) is the product of the draw ratio *@* (*@* = Δ*l*_2_/Δ*l*_1_, eqn (5)) and the starting velocity *υ*_1_ (Δ*l*_1_ = *υ*_1_*t*) of the jet. Therefore,7
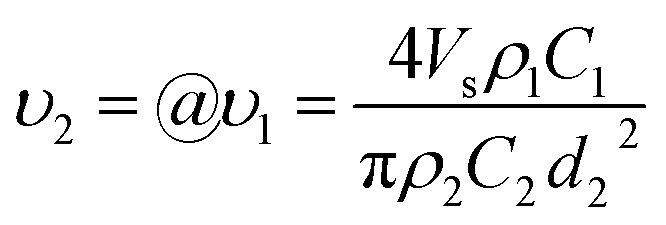
where the definitions and units of *C*, *V*_s_, *d*_2_ and *ρ* are the same as above. The values for *C*, *V*_s_ and *ρ* are easy to determine. The fiber diameter *d*_2_ can be measured using a scanning electron microscope (SEM) or an atomic force microscope (AFM). However, if the distance between the pendant drop and grounded collector is too large, the solvent will be evaporated to produce a dry nanofiber before it lands on the collector and the diameters will be no longer changed, which lead to an untrue jet velocity of fibers arriving on the collector. Therefore, if we want to know the real velocity, we must collect the fibers before the fibers are completely solidified.

Eqn (7) can also be deduced from another aspect. If we similarly assume that electrospinning under a strong electric field is an uninterrupted single-jet process at all times and that the polymer is consumed completely through the virtual micro-nozzle with a diameter *d*, then the consumption speed *Q*_p_ (g) of the polymer can be obtained from the parameters of the velocity of the charged bending fiber jet (*υ*_2_, m s^−1^), the density of the charged bending fiber jet (*ρ*_2_, g cm^−3^), the percentage of polymer in the bending fiber jet (*C*_2_), and the cross-sectional area of the final nanofiber jets,

8 *Q*_P_ = *ρ*_2_(*υ*_2_*tS*_2_)*C*_2_

where *t* is the spinning time and *S*_2_ is the cross-sectional area of the micro-nozzle.

The consumption of polymer can also be derived from the parameters of consumption of the spinning solution (*Q*_s_, mL), the density of the straight segment jet (*ρ*_1_, g cm^−3^), and the concentration of the polymer solution (*C*_1_), expressed as the product of the solution concentration *C* and solution volume, which is given by:

9 *Q*_P_ = *ρ*_1_*Q*_s_*C*_1_

The flow rate of the spinning solution *V*_s_ can be replaced by *Q*_s_/*t*, and *S*_2_ is equal to π(*d*_2_/2)^2^. Combining eqn (8) and (9), the velocity of the nanofibers *υ*_2_ can be given by:10
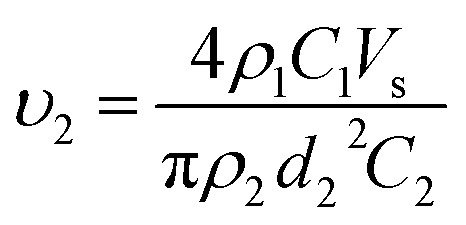
which is the same as that of eqn (7).

Based on our calculations and derivations, we found that the main unreasonable aspect in ref. 38 was the velocity of the jet at a diameter of 100 μm. When the flow rate of the polymer solution was 1 mL h^−1^, according to eqn (3), the velocity of the jet with a diameter of 100 μm was 0.0353 m s^−1^ rather than 1 m s^−1^. Putting the parameters of the initial jet velocity of 0.0353 m s^−1^, diameter of 100 μm, final diameter of the fibers of 200 nm density of 1.3 g cm^−3^, and the solution concentration of 0.06 g mL^−1^ into eqn (7) gives the final velocity of the nanofibers as 408 m s^−1^, which is far smaller than 60 000 m s^−1^.^38^

### 3.5. Electrospinning of PAA solution

**Fig. 2:**
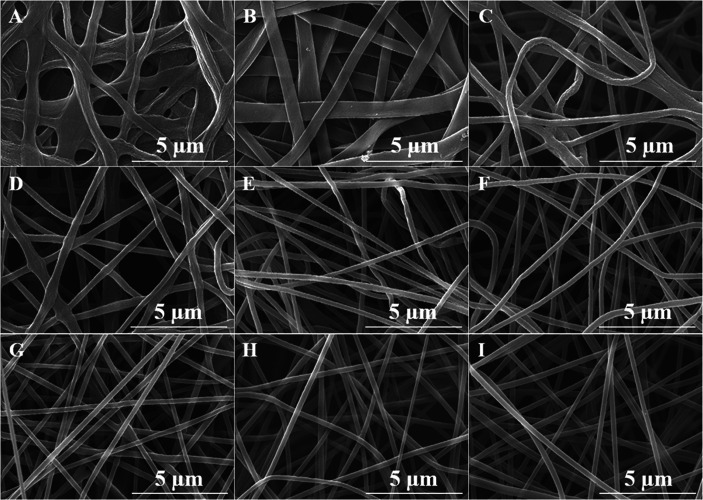
SEM images of PAA nanofibers collected in the water with different collecting distances of (A) 6 cm, (B) 8 cm, (C) 10 cm, (D) 11 cm, (E) 13 cm, (F) 15 cm, (G) 20 cm, (H) 25 cm and (I) 30 cm, respectively.

PAA could immediately be precipitated by dropping PAA solution into water. Herein, in order to get detailed information about the changes in the jet diameter during electrospinning, a water bath is used to collect nanofibers at different collecting distances. The corresponding fiber morphology and the fiber diameter are shown in Fig. 2 and Table 2, respectively. Ribbon-like fibers are formed when the collecting distance is below 8 cm. When the collecting distance is too short, there is still too much solvent in the fibers (more than 90 wt%, Table 2), leading to the collapse of the fiber jets.^57,58^ The widths of the belts are about 640 nm and 580 nm, which were assumed to be the diameter of the fiber jets at collecting distances of 6 cm and 8 cm, respectively. As the collecting distance increased, the resultant fibers become round and uniform, and the diameter of the fiber jets becomes smaller. When the collecting distance is longer than 15 cm, due to the complete solidification of the jet, the diameter of the resultant PAA fibers are almost constant at around 245 nm.

**Table 2:** Diameter, amount of residual solvent, density, draw ratio and velocity of jets at different distances

Collecting distance (cm)	Diameter of jet (nm)	Amount of solvent (%)	Density of jet (g cm^−3^)	Draw ratio^a^	Velocity^b^ (m s^−1^)	
					*υ*_1_	*υ*_2_
0	18 800	96.50	0.9504	—	0.998	
6	640 ± 132	91.40	0.9611	347		346
8	580 ± 155	90.39	0.9632	378		377
10	345 ± 106	88.24	0.9677	869		867
11	325 ± 87	85.68	0.9731	799		797
13	256 ± 31	74.36	0.9968	703		701
15	260 ± 29	70.38	1.0052	585		584
20	247 ± 17	53.76	1.0401	401		400
25	243 ± 22	26.86	1.0965	248		248
30	245 ± 15	25.45	1.0995	239		239

a Draw ratio was calculated from eqn (5).

b Velocity *υ*_1_ was calculated from eqn (1) and *υ*_2_ was calculated from eqn (7).

The densities of polymer PAA (BPDA-PDA) and solvent DMAc are 1.153 g cm^−3^ and 0.943 g cm^−3^, respectively. The concentration of the PAA electrospinning solution is 3.5 wt% and the flow rate of the solution is 1 mL h^−1^. According to eqn (7), the velocity of the final nanofiber jet *υ*_2_ can be calculated by:
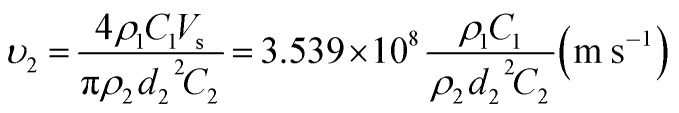
where the unit of *d*_2_ is nm, and the unit of *υ*_2_ is m s^−1^. The density of the charged jet can be calculated by

12 *ρ*_2_ = *φ*_DMAc_*ρ*_DMAc_ + *φ*_PAA_*ρ*_PAA_

where *φ*_DMAc_ and *φ*_PAA_ are the amount (wt%) of residual solvent DMAc and polymer PAA in the charged jet, respectively. The amount of residual solvent DMAc can be calculated from the weight before and after drying the fibers. The density of the charged straight segment jet equal to the solution with a concentration of 3.5 wt%, is 0.9504 g cm^−3^. Therefore, the draw ratio is calculated by *@* = *υ*_2_/*υ*_1_, where the initial velocity of jet *υ*_1_ is 1 m s^−1^. The jet diameter and velocity were changed with different collecting distances. The velocities and draw ratios at different collecting distances are listed in Table 2. As shown in Fig. 2, when the collecting distance increased, the diameter of the nanofiber jet was decreased; however, the velocity of the nanofiber jet was first increased and then decreased. The increased velocity of the nanofiber jet is in accordance with the acceleration process during electrospinning. When the collecting distance was larger than 10 cm, the velocity of the nanofiber jet decreased, which could be due to the resistance from air and the accelerated solidification of the fiber jet. Generally, the velocity of the final fiber jet is at the order of 100 m s^−1^, which is far lower than the value of 60 000 m s^−1^ from a previous report.^38^ Such a high velocity value in the previous report could be attributed to an overestimated initial jet diameter of 100 μm and the velocity of the straight segment at 1 m s^−1^. First, according to eqn (2), if the flow rate of the spinning solution is 1 mL h^−1^, the velocity of the jet at a diameter of 100 μm will not be 1 m s^−1^ but 0.0353 m s^−1^. Second, according to eqn (3), if the initial flow rate is 1 mL h^−1^, the jet diameter will be 18.8 μm and the initial jet velocity will be 0.998 m s^−1^. Then, the draw ratio of the jet can be calculated and is listed in Table 2. When the collecting distance was 10 cm, the draw ratio of the jet was 869, which was highest among other collecting distances, but much lower than the draw ratio of 60 000 in the previous report.^38^ Considering that the eqn (7) is deduced under ideal conditions without the consideration of the resistant force of air and other possible factors, the actual value of the draw ratios and the velocities of the charged jets would be lower than those calculated in Table 2.

## 4. Conclusion

Simple and reasonable models were successfully constructed to predict the draw ratio and velocity of the charged polymer solution jet by considering the evaporation of solvent and the solidification of the jet. Further calculations based on the model experiment proved that the electrically charged polymer solution jet first experienced an acceleration process, followed by a deceleration process due to the stretching of the electric force and the resistance force from air, respectively. The calculation based on the experiment revealed that the draw ratio and the velocity of the charged jet was lower than 350 and 1000 m s^−1^, respectively, both of which were much lower than those (draw ratio of 60 000 and velocity of 60 000 m s^−1^) in the previous reports.

## Conflicts of interest

The authors declare no conflict of interest.

## Supplementary Material
